# Anti-Inflammatory Effects of IKK Inhibitor XII, Thymulin, and Fat-Soluble Antioxidants in LPS-Treated Mice

**DOI:** 10.1155/2014/724838

**Published:** 2014-06-19

**Authors:** E. G. Novoselova, M. O. Khrenov, O. V. Glushkova, S. M. Lunin, S. B. Parfenyuk, T. V. Novoselova, E. E. Fesenko

**Affiliations:** Institute of Cell Biophysics of Russian Academy of Sciences, Pushchino, Moscow, Russia

## Abstract

The present study was designed to compare the anti-inflammatory effects of several agents applied *in vivo*, namely, a synthetic inhibitor of the NF-*κ*B cascade, fat-soluble antioxidants, and the thymic peptide thymulin. Cytokine response in LPS-treated mice was analysed in tandem with the following parameters: the synthesis of inducible forms of the heat shock proteins HSP72 and HSP90*α*; activity of the NF-*κ*B and SAPK/JNK signalling pathways; and TLR4 expression. Inflammation-bearing Balb/c male mice were pretreated with an inhibitor of IKK-*α*/*β* kinases (IKK Inhibitor XII); with thymulin; with dietary coenzyme Q_9_, *α*-tocopherol, and *β*-carotene; or with combinations of the inhibitor and peptide or antioxidants. Comparable anti-inflammatory effects were observed in inflammation-bearing mice treated separately with thymulin or with dietary antioxidants administered daily for two weeks before LPS treatment. When LPS-injected mice were treated with the inhibitor and antioxidants together, neither plasma cytokines, signal proteins, nor heat shock proteins recovered more efficiently than when mice were treated with these agents separately. In contrast to antioxidant diet, the thymulin was shown to increase the effect of IKK Inhibitor XII in preventing IKK activation in LPS-treated mice.

## 1. Introduction

The nuclear factor kappa B (NF-*κ*B) family consists of transcription factors activated in response to a variety of cellular stressors [1]. The transcription factor NF-*κ*B is a key component of the cellular response to damage, stress, and inflammation [2, 3]. The NF-*κ*B proteins consist of 5 subunits, which bind to DNA as a dimer; the most common of these subunits is the p65/p50 heterodimer. NF-*κ*B activation via the canonical pathway is mediated by inhibitory kappa B (I*κ*B) kinase (IKK), which is activated in response to a variety of pathogenic factors [4]. Activated IKK phosphorylates an inhibitory protein, I*κ*B, which results in I*κ*B degradation and thereby allows NF-*κ*B to translocate to the nucleus and to induce the synthesis of proinflammatory molecules. Studies that have utilised a direct blockade of the enzymatic activities of IKK, JNK, or p38 MAPK have demonstrated the great potential for such treatments to elicit anti-inflammatory effects. Selective inhibitors for different signal pathways, including the NF-*κ*B cascade, are therefore potentially useful in the treatment of inflammation. We have recently reported that synthetic inhibitors of Toll-like receptor 4 (TLR-4), stress-activated protein kinase JNK (SAPK/JNK), and NF-*κ*B signalling (CLI-095, SP600125, and IKK Inhibitor XII, resp.) reduced the* in vitro* effect of LPS on macrophage-like RAW 264.7 cells. Among these three studied inhibitors, the suppressor of the NF-*κ*B cascade, IKK Inhibitor XII, has been shown to be the most effective antitoxic agent* in vitro* [5].

The first aim of present study was to determine whether IKK Inhibitor XII is effective* in vivo* and how the effectiveness of this inhibitor is affected when used in combination with other anti-inflammatory agents. To address these knowledge gaps, we used a mouse model of acute septic inflammation induced by LPS from the Gram-negative bacteria* Escherichia coli*. Numerous studies have reported an increase in the production of reactive oxygen species during sepsis. It is well known that mutual cross-talk exists between reactive oxygen species and NF-*κ*B signalling [6]. Since antioxidants act not individually, but synergistically, a mixture of vitamin E, ubiquinone Q_9_, and *β*-carotene was added to the animal diet. In addition, in an attempt to gain more insight on the effects of each fat-soluble antioxidant, we studied coenzyme Q_9_, *α*-tocopherol, and *β*-carotene in cellular model. Earlier we have shown that oxidative stress linked with acute inflammation can be prevented by the mixed dietary fat-soluble antioxidants coenzyme Q_9_, *α*-tocopherol, and *β*-carotene [7]. The second aim of the present study was to test if these antioxidants can reinforce IKK Inhibitor XII activity.

Finally, the third aim of study was screening the anti-inflammatory activity of IKK Inhibitor XII in combination with the thymic peptide thymulin. The activity of this peptide has been reported not only in the thymus but also in the bloodstream, affecting extrathymic immune cells [8]. At present, the role of thymic peptides in the control of septic inflammation is unclear. It has been shown that thymic peptides can affect signalling pathways during both normal conditions and systemic inflammation [9]. Other authors believe that the mechanisms of the anti-inflammatory effects of thymic peptides, including thymulin, may involve NF-*κ*B and p38 signalling cascades, which play crucial roles in inflammation [10, 11]. Recently we have demonstrated that thymulin* in vitro* and* in vivo* affected the NF-*κ*B cascade in tandem with the production of proinflammatory cytokines, nitric oxide, and heat shock proteins in immune cells [12, 13]. We hypothesised that the antioxidants and/or thymulin could act as adjuvants, strengthening the anti-inflammatory effect of IKK Inhibitor XII.

## 2. Materials and Methods

### 2.1. Protocol of Experiments

**Figure 1:**
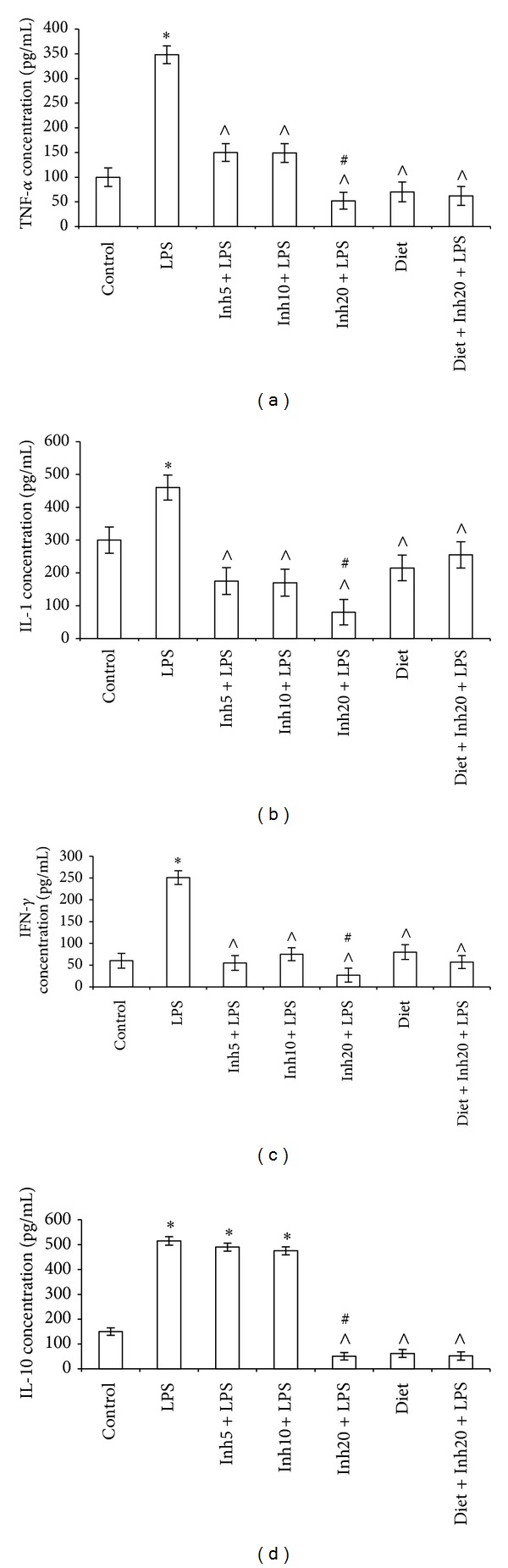
Plasma cytokine values in inflammation-bearing mice treated with antioxidant-rich diet and IKK Inhibitor XII. Seven mouse groups were used: inflammation-bearing (IB) mice; three groups of IB mice pretreated with inhibitor at concentration 5 mg/kg (Inh5), 10 mg/kg (Inh10), or 20 (Inh20) mg/kg; IB mice pretreated with antioxidant-rich diet; IB mice pretreated with antioxidant-rich diet plus inhibitor; and untreated controls. Each group consisted of 4 mice, which was examined individually. Each value is average mean ± SD from four independent experiments; the measurements were made for each individual mouse in six duplicates. Data are expressed in pg/mL of plasma. *Significantly different from control, *P* < 0.05. ^∧^Significantly different from LPS-group, *P* < 0.05. ^#^Significantly different from Inh(5) or Inh (10) plus LPS-group, *P* < 0.05.

**Figure 2:**
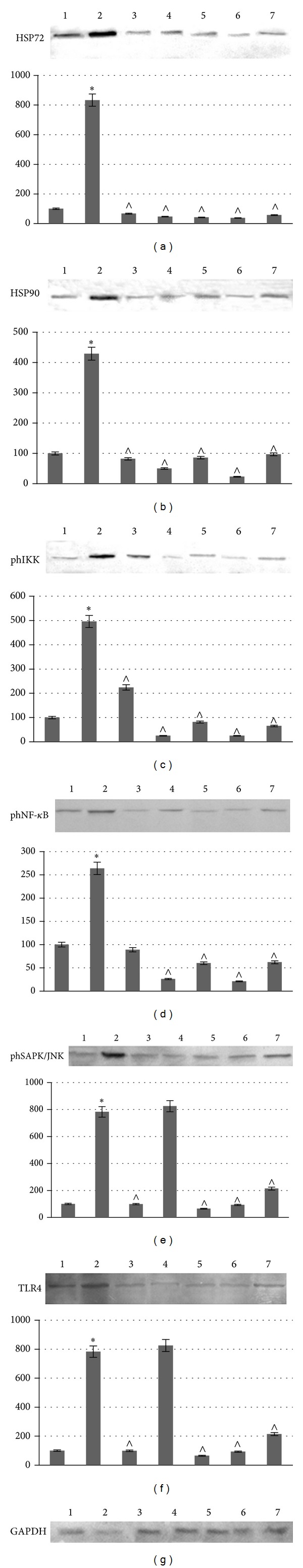
The effects of IKK Inhibitor XII and antioxidant-rich diet on phosphorylation of RelA, IKK, and SAPK/JNK and on the expression of TLR4 and heat shock proteins in the splenic lymphocytes from inflammation-bearing mice. The animal's groups that was indicated in Figure 1 were used (1, control; 2, IB mice; 3, IB + Inh5; 4, IB + Inh10; 5, IB + Inh20; 6, IB + diet; 7, IB + diet + Inh20). Western blot analysis of extracts from isolated mice lymphocytes was provided using corresponding antibodies or anti-GAPDH antibody (bottom). Blot pictures show a single representative experiment from four independent experiments. Histograms below protein bands show protein levels calculated as mean relative units correspondingly to internal control and are the results of blots densitometry by program QAPA from four independent experiments. *Significantly different from control, *P* < 0.05. ^∧^Significantly different from LPS-group, *P* < 0.05.

**Figure 3:**
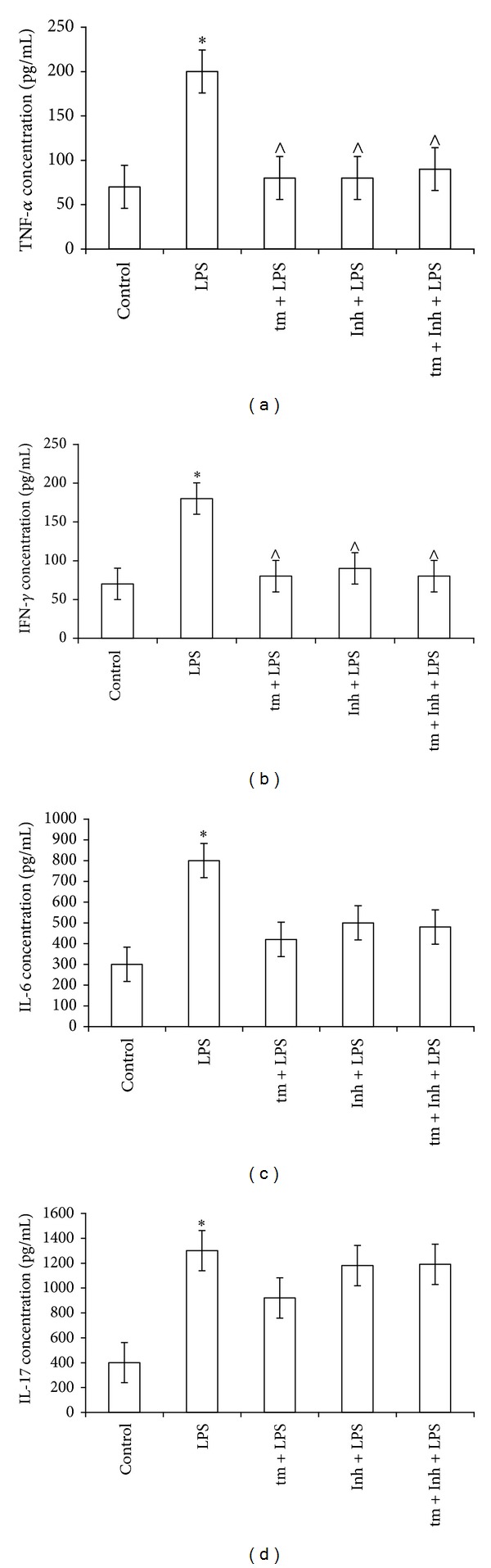
Plasma cytokine values in inflammation-bearing mice treated with thymulin and IKK Inhibitor XII. Five mouse groups were used: IB mice; IB mice pretreated with 20 mg/kg inhibitor; IB mice pretreated with thymulin; IB mice pretreated with thymulin plus inhibitor; and untreated controls. Each group consisted of 4 animals, which were examined individually. Each value is average mean ± SD from four mice; the measurements were made for each individual mouse in six duplicates. Data are expressed in pg/mL of plasma. *Significantly different from control, *P* < 0.05. ^∧^Significantly different from LPS-group, *P* < 0.05.

**Figure 4:**
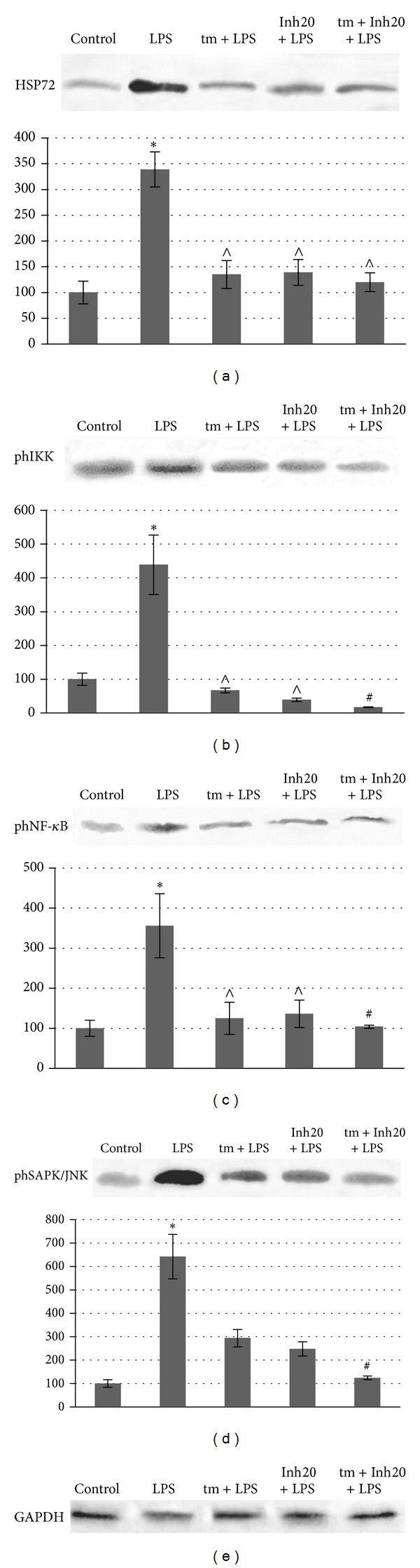
The effects of IKK Inhibitor XII and thymulin on phosphorylation of RelA, IKK, SAPK/JNK, and expression of Hsp72 in the splenocytes from inflammation-bearing mice. The animal's groups that indicated in Figure 3 were used. Western blot analysis of extracts from isolated mice lymphocytes was provided using corresponding antibodies or anti-GAPDH antibody as loading control (bottom). Blot pictures show a single representative experiment for four independent experiments. Histograms below protein bands show protein levels calculated as mean relative units correspondingly to internal control and are the results of blots densitometry by program QAPA from four independent experiments. *Significantly different from control, *P* < 0.05. ^∧^Significantly different from LPS-group, *P* < 0.05. ^#^Significantly different from (Inh+LPS)-group, *P* < 0.05.

Two independent series of* in vivo* experiments were performed. The first series included seven groups, with each group consisting of 4 mice, and the results of the study are shown on Figures 1 and 2. The second series performed at another point of time, included five groups, with each group consisting of 4 mice, and the results of the study are shown on Figures 3 and 4.

### 2.2. Animals, Animal Inflammation Model, Diet, Thymulin, and Inhibition of the NF-*κ*B Cascade

Male Balb/c 8- to 10-week-old (25–27 g) mice were maintained under standard laboratory conditions (20–21°C, 10–14 h light-dark cycle, and 65% humidity) with food and water available* ad libitum*. The standard food pellets contained a balanced diet with proteins, vitamins, and minerals. The procedures followed were approved by the ethics committee of the institution and were in accordance with the Guidelines for Ethical Conduct in the Care and Use of Animals. A diet enriched with *β*-carotene (2 mg/kg body weight), *α*-tocopherol (2 mg/kg body weight), and ubiquinone Q_9_ (8 mg/kg body weight) was administered daily for 15 days prior to LPS treatment. All antioxidants were purchased from Sigma, USA. The antioxidant formula used took into account the approximate levels of these antioxidants in the animals' tissues [14–16], such that each compound did not exceed its physiological level in mice. The antioxidant mixture was stirred into 2 g of pellets and was given daily before feeding at 8 am; antioxidants were fed to each mouse individually. Before morning feeding, animals in the other groups were fed pellets without antioxidants.

Inflammation was induced by a single intraperitoneal injection of lipopolysaccharide (LPS) from* Escherichia coli* (Serotype 026.B6, “Sigma,” USA) (2.5 mg per kg body weight). Thymulin solution (1.5 mg/kg) was injected intraperitoneally 1.5 hr before LPS treatment and was prepared from serum thymic factor (American Peptides, USA), to which an equimolar concentration of ZnCl_2_ was added [17]. IKK Inhibitor XII, at concentrations ranging from 5 to 20 mg/kg was injected intraperitoneally 1 h prior to LPS treatment. Mice were decapitated 6 h after LPS injection in parallel with the corresponding control groups. All measurements were carried out individually for each mouse, with nine replicates.

### 2.3. Blood Plasma and Cells

Plasma was isolated from blood collected during the decapitation of animals. The blood samples were kept for 3–5 h at 4°*С* and centrifuged at 200 g; supernatants were then collected for cytokine assays. Lymphocytes from the spleen were isolated in 199 medium (Sigma, USA) containing 1% 1 M HEPES solution, 100 *μ*g/mL streptomycin, and 10% fetal bovine serum. Erythrocytes were lysed in Tris-buffered ammonium chloride (0.01 M Tris-HCl with 0.15 M NaCl—0.83% NH_4_Cl, 9 : 1). After washing, the samples were stored at a concentration of 1 × 10^8^ cells/mL in RPMI 1640 medium at −20°C until Western blotting was performed.

### 2.4. ELISA

ELISA was used to determine the concentration of cytokines in blood plasma. ELISA Development Kits for mouse TNF-*α*, IL-1*α*, IL-6, IL-17, IL-10, and IFN-*γ* (Peprotech, USA) were used. To visualise binding, 100 *μ*L of ABTS green dye (Sigma, USA) dissolved in 0.05 M citrate buffer (pH 4.0) with 0.01% hydrogen peroxide was added. The optical density was measured at 405 nm with a plate spectrophotometer (Multiscan EX, Thermo Electron Corporation).

### 2.5. Western Blot Analysis

To prepare specimens, 1 × 10^8^ splenic cells were lysed using an ultrasonic disintegrator with constant stirring for 2 min. The total protein concentration of each sample was then determined by the Bradford method. The proteins in each sample were then precipitated in acetone, solubilised, boiled for 5 min, and stored at −70°C. Proteins were resolved by electrophoresis over a 10% PAGE gel and then transferred from the gel onto a nitrocellulose membrane (GE Healthcare, Amersham, UK) in a transblot chamber. After blocking, the membrane was exposed for 2 hr to antibodies against the following mouse proteins: HSP70 antibody (rabbit anti-mouse HSP 72, clone SPA-812, inducible form, StressGen), HSP90 antibody (rabbit anti-Hsp90*α* [Hsp86], StressGen), phospho-NF-*κ*B antibody (phospho-NF-*κ*B p65 [Ser 536], #3031, Cell Signaling Technology, Danvers, MA), rabbit phospho- IKK*α*/*β* antibody II (Ser 176/180 (Cell Signaling Technology, USA), rabbit phospho-SAPK/JNK antibody to synthetic phospho-peptide SAPK/JNK, or rabbit TLR4 antibody (#2246, Cell Signaling Technology, USA). After washing, the nitrocellulose membranes were incubated for 1 hr with the anti-rabbit biotinylated antibody (Jackson ImmunoResearch, West Grove, PA), and peroxidase-conjugated streptavidin was added for 1 hr. The loading control was a rabbit monoclonal antibody against a synthetic peptide near the carboxy terminus of human glyceraldehyde-3-phosphate dehydrogenase (GAPDH) (Cell Signaling). An ECL-plus chemiluminescent cocktail (Amersham/GE) was then used to develop the blots according to the manufacturer's instructions, and the blot was then exposed to film. Quantitative evaluation of protein bands was then performed using the Qapa computer program (Pushchino, Russia).

### 2.6. *In Vitro* Addition of Antioxidants

Fifty *μ*M ubiquinone, 10 *μ*M *α*-tocopherol, or 10 *μ*M *β*-carotene were added to the cultivating medium as water-alcohol emulsions (for Q_9_ and *α*-tocopherol) or a water-Tween 85 emulsion (for *β*-carotene). Samples with medium supplemented only with the equivalent amount of alcohol or Tween 85 served as controls. The cells were cultivated for 24 h at 37°C in a humidified atmosphere containing 5% CO_2_. In all cases, the final alcohol and Tween 85 concentrations did not exceed 1% in control or experimental samples.

### 2.7. Blood Glucose Measurements

Blood glucose levels were assayed in all groups of mice prior to manipulations and at 1, 2, and 6 h after LPS treatment. During glucose measurement, the tail tip (2-3 mm) was excised and massaged to harvest a small volume of blood (1.0–10 *μ*L) which was placed into the hole of a blood glucose test strip (Accu-Chek Performa, Germany). Glucose measurements were obtained using a rapid blood glucose meter (Accu-Chek Performa, Germany).

### 2.8. Statistical Analysis

Statistical analysis was performed using Statistica/Win 6.0 software (Tulsa, OK). One-way analysis of variance (ANOVA) followed by a post hoc Tukey test was used to determine the significance of differences among groups, with *P* values ≤ 0.05 considered significant. All values were expressed as means (±SE).

## 3. Results

Initial experiments were performed to reveal the abilities of each of used antioxidants (*β*-carotene, *α*-tocopherol, and ubiquinone Q_9_) to eliminate reactive oxygen species in murine peritoneal macrophages. It is well known that oxidative stress linked with LPS treatment results in proinflammatory cytokine response, including extremely TNF-*α* production [18]. So, we tested the protective effect of each of antioxidants by measuring the production of TNF-*α* level in LPS-treated macrophages as shown in Table 1.

The series of observations strongly indicated that separately the addition of the ubiquinone, *α*-tocopherol, and *β*-carotene to the culture medium* in vitro* significantly decreased the TNF production in LPS-treated macrophages. In addition, effect of three mixed compounds was rather more appreciable, indicating synergistic interaction between fat-soluble antioxidants.

In each experimental group, blood glucose levels decreased to 70%–80% compared to controls one hour after LPS treatment, but rapidly recovered to the control level, being unaltered at 2 and 6 h. In addition, no substantial differences in blood glucose levels were found between experimental groups (data not shown). We can assume a glucose-independent mechanism of anti-inflammatory effects of agents studied.

### 3.1. The Effects of Dietary Antioxidants and IKK Inhibitor XII on LPS-Treated Mice Immunity

#### 3.1.1. Plasma Cytokines in LPS-Treated Mice Presubjected to Dietary Antioxidants and IKK Inhibitor XII

To examine the amounts of proinflammatory molecules associated with acute inflammation and the protective effect of the inhibitor and diet, the plasma cytokine concentrations were measured concurrently in seven mouse groups treated with IKK Inhibitor XII, dietary antioxidants, or a combination thereof. Treatment with LPS alone resulted in an expected increase in plasma proinflammatory cytokines (e.g., IL-1*α*, IFN-*γ*, and TNF-*α*). There was more than 2-fold increase in plasma TNF-*α* levels and a more than 3-fold increase in IFN-*γ* levels (Figure 1).

Additionally, levels of the anti-inflammatory cytokine IL-10 increased by approximately 1.5-fold. This finding agrees with previously reported data that demonstrated that LPS* in vivo* increased IL-10 production in peritoneal mouse macrophages [7].

In mice pretreated with different concentrations of IKK Inhibitor XII, plasma cytokine values were significantly decreased, and the maximal effect was observed at a concentration of 20 mg/kg. The pretreatment of mice with dietary antioxidants affected cellular responses to LPS; in cells from mice treated with antioxidant-rich diet, plasma cytokine values were reduced (Figure 1). It should be noted that the effects of antioxidants on the inflammatory response measured by cytokine production were less suppressive than the effects induced by the IKK inhibitor; the difference, however, was statistically significant for IL-1*α*, IL-10, and IFN-*γ* values. Additionally, when mice were simultaneously exposed to the inhibitor and antioxidants, nonadditive effects from the IKK inhibitor and dietary antioxidants were observed.

#### 3.1.2. Signal and Stress Proteins in LPS-Treated Mice Presubjected to Dietary Antioxidants and IKK Inhibitor XII

To examine the extent of NF-*к*B activation associated with inflammation, the levels of the phosphorylated dimer p65/RelA and phosphorylated IKK-*α*/*β* were measured by the immunoblotting of splenic lymphocytes. There was a more than 2-fold increase in phosphorylated p65, which correlated with a significant (more than 7-fold) increase in the level of phosphorylated IKK in these cells (Figure 2). The pretreatment of mice separately either with the inhibitor or with dietary antioxidants significantly decreased the phosphorylation of both p65 and I*к*B, and the combination of the inhibitor and antioxidants did not result in an additive effect.

To elucidate the role of an essential receptor for LPS and TLR4, we analysed cellular TLR4 levels using Western blot analysis. As shown in Figure 2, the addition of LPS induced an expected significant increase in TLR4 levels in splenic cells. However, in the cells of mice pretreated with IKK Inhibitor XII or with antioxidants, these increases in TLR4 expression were completely ablated, indicating that the TLR4 pathway can be interregulated via NF-*к*B signalling as well as by the redox balance.

Additionally, to assess the level of stress response in LPS-treated mice, the expression of two inducible heat shock proteins, Hsp72 and Hsp90-*б*, was studied in splenic lymphocytes. LPS induced a 4-fold rise in Hsp90 expression and a more than 8-fold increase in Hsp72 production (Figure 2). Again, the pretreatment of mice with IKK Inhibitor XII or with antioxidants resulted in the complete ablation of Hsp72 and Hsp90-*α* spikes in cells after injection with LPS. Additionally, these two simultaneously applied treatments did not have a reciprocal interaction, as demonstrated by the measurement of both heat shock proteins, of cytokines, and of signalling proteins.

### 3.2. The Effects of Thymulin and IKK Inhibitor XII on LPS-Treated Mice Immunity

#### 3.2.1. Plasma Cytokines in LPS-Treated Mice Subjected to Thymulin and IKK Inhibitor XII

To determine whether thymulin with or without IKK Inhibitor XII can downregulate the* in vivo* response to LPS, the values of plasma IL-6, IL-17, IFN-*γ*, and TNF-*α* were measured in LPS-injected mice using five mouse groups indicated in the legend to Figure 3. Note that other panel of cytokines was tested in these experiments based on our previous data on thymulin activity. Indeed, these cytokines are predisposed to thymulin control [19].

Based on the above results, the optimal concentration of 20 mg/kg inhibitor was used in this experimental series examining the combined effect of thymulin with IKK Inhibitor XII. These experiments confirmed that an increase in plasma TNF-*α* levels occurs after LPS treatment, supporting the results of the experiments displayed in Figure 1. The pretreatment with thymulin or with IKK Inhibitor XII decreased the accumulation of the TNF-*α* in plasma. The concentration of plasma TNF-*α* in mice pretreated with the inhibitor and thymulin together did not significantly differ from that measured in LPS-treated mice. There was a more than 3-fold increase in plasma IFN-*γ* in mice treated only with LPS; both IKK Inhibitor XII and thymulin prevented this increase. The combined effect of the inhibitor and thymulin in preventing LPS-mediated increases of plasma IFN-*γ* was no greater than the individual activity of each agent.

In these experiments, we detected a significant increase in the concentration of IL-6 in LPS-treated mice, which was partly but not significantly decreased by pretreating the mice with IKK Inhibitor XII or with thymulin, demonstrating that these treatments could not completely ablate plasma IL-6 levels. It is important to note that when these two agents were administered together, IL-6 levels were still not completely normalised. In addition, there was a more than 3-fold increase in plasma IL-17 from LPS-treated mice, but the inhibitor and thymulin, alone or in combination, could not prevent the increase in IL-17 (Figure 3).

#### 3.2.2. Signal and Stress Proteins in LPS-Treated Mice Subjected to Thymulin and IKK Inhibitor XII

Similar to results from the first experimental series, the treatment of mice with LPS resulted in the increased phosphorylation of RelA and IKK (Figure 4).

Thymulin and particularly IKK Inhibitor XII reduced IKK phosphorylation. Interestingly, compared to separate treatments with peptide or with inhibitor, the combined treatment with thymulin and the inhibitor resulted in an apparent increase in the protective effect directed towards the prevention of excessive IKK phosphorylation. It has been previously shown that IKK Inhibitor XII* in vitro* affects the SAPK/JNK pathway in RAW 264.7 cells [5]. To examine the* in vivo* effects of the inhibitor and thymulin on the activity of SAPK/JNK signalling, the levels of phosphorylated SAPK/JNK were measured in lymphocytes from 5 animal groups. In contrast to linear RAW 264.7 cells, which generate two phosphorylated isoforms, JNK1 and JNK2,* ex vivo* cells expressed only a single form of phosphorylated SAPK/JNK. LPS induced an approximately 7-fold increase in SAPK/JNK phosphorylation, and in mice separately treated with thymulin or with IKK Inhibitor XII, the spike in phosphorylated SAPK/JNK significantly decreased. It should be noted that the combined treatment with thymulin and inhibitor resulted in a summation of their effects and the normalisation of SAPK/JNK phosphorylation.

There was also a reduction in cellular Hsp72 expression in cells from inflammation-bearing mice that were pretreated with thymulin or the inhibitor (Figure 4). Collectively, these data demonstrate that the pharmacological inhibition of IKK/NF-*к*B and SAPK/JNK activation leads to the attenuation of LPS-related inflammation.

## 4. Discussion

Over the last decade, an inhibitor of the NF-*к*B signalling pathway, IKK Inhibitor XII, has been discovered and investigated for its clinical potential in inflammatory diseases [18]. This inhibitor is a synthetic small hydrophobic molecule that can freely penetrate into the cell. IKK Inhibitor XII, a cell-permeable amino-diarylbenzamide compound, acts as a potential ATP site targeting inhibitor against IKK-1 and IKK-2. Interestingly, IKK Inhibitor XII, a selective inhibitor of NF-*к*B signalling that functions by decreasing NF-*к*B phosphorylation and thus reducing the probability of translocation of the NF-*к*B into the nucleus, also prevented SAPK/JNK activation in LPS-treated cells (Figure 2). These results coincide with findings demonstrating that SAPK/JNK signalling is regulated by the NF-*к*B pathway [20, 21]. Indeed, it is generally suggested that NF-*к*B activation plays a critical role in LPS-induced responses and that the suppression of NF-*к*B pathway activity prevents its ability to activate the transcription of a wide variety of genes encoding immunologically relevant proteins. It is also known that targeting IKK also may affect other pathways in addition to NF-*к*B. For example, IKK affects p53, FOXO3A, and HIF-1 in addition to NF-*к*B [22, 23].

We demonstrated that the pharmacological inhibition of the IKK/NF-*к*B pathway by IKK Inhibitor XII delays the proinflammatory response in LPS-treated mice. Thus, many processes are shown to be altered in mice undergoing inflammation, including increases in cytokine production, the stimulation of heat shock proteins and TLR4 expression, and the activation of NF-*к*B and SAPK/JNK signalling, were at least partially corrected* in vivo* by NF-*к*B inhibition. This finding provides additional experimental evidence that an increase of NF-*к*B activity plays a causal role in LPS-related inflammation.

Our data show that a diet supplemented with antioxidants prevents NF-*к*B activation in mice treated with LPS. It should be noted that several previous studies using similar animal inflammation models demonstrated that pretreatment with antioxidant N-acetylcysteine or N-acetylcysteine combined with *α*-tocopherol before endotoxin administration resulted in a decrease in NF-*к*B activation [24, 25], lowered TNF-*α* release, and increased survival [26]. In addition, the administration of N-acetylcysteine with vitamin E and *β*-carotene reduced lipid peroxidation and restored GSH levels in endotoxic rats [27]. Moreover, it was demonstrated that the genetic reduction of NF-*к*B reduced the amount of mitochondrial-derived ROS [28].

In the present study, we hypothesised that an excess of coenzyme Q in the diet, along with other fat-soluble vitamins, could be very useful due to the unique properties of ubiquinones as suppressors of cholesterol synthesis, thereby enhancing membrane fluidity. A significant decrease in cholesterol synthesis and a subsequent drop in the cholesterol concentration of the cellular membranes were observed in rats fed a diet supplemented with ubiquinone Q_9_. Additionally, the inhibition of cholesterol synthesis in lymphocytes cultivated* in vitro* with coenzyme Q_9_ was demonstrated [29, 30]. Interestingly, our data indicated that the preintervention with dietary antioxidants decreased the magnitude of the inflammatory response, but diet did not amplify the effect of IKK Inhibitor XII. These results demonstrating nonadditive anti-inflammatory effects of IKK Inhibitor XII and dietary antioxidants suggest antioxidant activity of the studied inhibitor. Thus, it can theoretically be assumed that there was a coincidental interaction between the protective mechanisms of the inhibitor and antioxidants, which may both be mediated via the reduction of reactive oxygen species. This assumption is consistent with recent data demonstrating that the inhibition of NF-*к*B activity reduces oxidative stress and damage* in vitro* and* in vivo* [28].

Thymic peptide thymulin, which is a metallopeptide consisting of a nonapeptide (Glu-Ala-Lys-Ser-Gln-Gly-Gly-Ser-Asp) that couples with zinc ions, mostly has inhibitory effects on immune responses [12, 31] and also stimulates endocrine systems, indicating its role in the recovery from inflammatory conditions [32]. Using thymulin as an immune-modulator in the second experimental line, we tested a repertoire of cytokines, including IL-6 and IL-17. Although IL-17 was identified more than 15 years ago as a product of CD4^+^ T cells, it was only recently proven that IL-17 is preferentially produced by a subset of T cells, specifically Th17, which have been shown to be very important in the pathogenesis of autoimmune disease. Additionally, it has been shown recently that thymulin significantly reduces the disease severity in mice with acute experimental autoimmune encephalomyelitis [33]. IL-17 and IL-6 are important in many disorders characterised by immune self-recognition, and IL-6 is known to induce the differentiation of Th17 cells [19, 34], but the role of Th17 cells in septic inflammation is still unclear. We demonstrated that the separate administration of the inhibitor and thymulin or their combination did not significantly change the plasma IL-17 concentration. Other cytokines, namely, TNF-alpha and IFN-gamma, were significantly decreased in mice that were pretreated with thymulin, IKK Inhibitor XII, or the combination thereof. These data support the assumption that Th17 cells do not play an active role in inflammatory pathogenesis induced by LPS. The plasma cytokine observations certainly do not provide a complete picture regarding the response to damage. It is possible that the release of cytokines is temporally differentiated, and the extent of release and utilisation seems to be nonsynchronous for different cytokines. Indeed, it was reported that after the intravenous administration of endotoxin in humans, plasma IL-6 increased slowly compared with plasma TNF-alpha [35, 36].

Alternately, monitoring short-lived signal and stress proteins in the cells enables a more complete evaluation of cellular responses. Indeed, inhibition of the IKK/NF-*к*B pathway using IKK Inhibitor XII delayed phosphorylation of both independent steps of NF-*к*B signalling, IKK, and p65. In addition, activation of the SAPK/JNK pathway also decreased in cells from LPS-plus-inhibitor-treated mice. The results of the present study demonstrate that thymulin enhances the activity of the IKK Inhibitor XII. Taken together, these results demonstrate that reducing NF-*к*B activity using both thymulin and IKK Inhibitor XII decreases the inflammatory response* in vivo*. The inhibition of NF-*к*B using thymulin plus NF-*к*B inhibitor offers what we believe to be a novel strategy for delaying or attenuating inflammatory diseases in patients with SIRS (systemic inflammatory response syndrome) or sepsis.

## Figures and Tables

**Table 1:** Effect of *in  vitro* added antioxidants on TNF-*α* production in LPS-treated peritoneal macrophages from mice.

Control 1	Control 2	LPS	LPS + Q_9_	LPS + *β*-carotene	LPS + *α*-tocopherol	LPS + antioxidant's mixture
20.2 ± 2.5	23 ± 2.6	80.1 ± 9.8^a^	32.4 ± 4.0^b^	35.2 ± 4.1^ab^	29.5 ± 3.1^b^	18.1 ± 2.5^bc^

Macrophages were cultivated for 24 h; 50 *μ*M of ubiquinone, 10 *μ*M *α*-tocopherol, and 10 *μ*M *β*-carotene were added in cultivating medium as water-alcohol emulsions (for Q_9_ and *α*-tocopherol) or water-Tween 85 emulsion (for *β*-carotene). The samples with only medium supplemented with the equal amounts of alcohol or Tween 85 served as controls (control 1 and control 2, correspondingly); then 2.5 *µ*g/mL lipopolysaccharide (LPS) from *Escherichia coli* was added to cells. TNF-*α* concentration shown in pg/mL was measured in the cell-free supernatants by ELISA kit. The final alcohol and Tween 85 concentrations did not exceed 1% in control or experimental samples. Each of values is the average mean ± S.D. from 12 duplicates.

^a^Significantly different from control, *P* < 0.05; ^b^significantly different from LPS-treated cells, *P* < 0.05; ^c^significantly different from LPS + Q_9_, LPS + *β*-carotene, and LPS + *α*-tocopherol-treated cells, *P* < 0.05.
